# Genetic Influences Affecting Alcohol Use Among Asians

**Published:** 1995

**Authors:** Tamara L. Wall, Cindy L. Ehlers

**Affiliations:** Tamara L. Wall, Ph.D., is an assistant professor in the Department of Psychiatry at the University of California, San Diego, California, and an adjunct member in the Department of Neuro-pharmacology at the Scripps Research Institute, La Jolla, California. Cindy L. Ehlers, Ph.D., is an associate member in the Department of Neuropharmacology at the Scripps Research Institute, La Jolla, California

**Keywords:** alcohol flush reaction, Asian, aldehyde dehydrogenase, isoenzyme, gene, genotype, risk factors, brain, AOD use, AOD dependence, ethanol metabolism, racial differences

## Abstract

Aldehyde dehydrogenase (ALDH) is one of the two enzymes primarily involved in alcohol metabolism. Several variants exist of the gene that produces ALDH. One of these gene variants, which generates a nonfunctional enzyme, is present in Asians but not in Caucasians and African-Americans. People with two copies of the defective gene respond to alcohol consumption with intense flushing and other unpleasant reactions, such as nausea. Consequently, these people consume very little alcohol and are at a much lower risk for alcoholism than people with functional ALDH genes. People with one copy of the defective gene also flush after ingesting alcohol and are at relatively lower risk for alcoholism than people with fully functional genes. In addition, these people have more intense, but not necessarily less pleasant, reactions to alcohol as assessed by both physiological and psychological measures. People with the defective gene variant also respond to alcohol consumption with characteristic changes in brain activity.

Alcoholism^1^ is a complex disorder arising from both biological (i.e., genetic) and sociocultural (i.e., environmental) factors. During the past two decades, studies in humans assessing individual differences in the response to alcohol have increased our understanding of how genetic traits may contribute to a person’s predisposition to alcoholism (Schuckit 1994). Some studies have aimed at identifying factors that might increase the risk for the disorder, whereas others have addressed protective factors. Most studies have focused on people who statistically are at high risk for alcoholism, such as children of alcoholic fathers, although some research has examined people with a statistically lower risk, such as those of Asian heritage. This article presents evidence that both alcohol use and alcoholism among Asians are genetically influenced, reviews studies examining individual differences in the response to alcohol that are associated with the genetic vulnerability for alcoholism, and provides a theoretical perspective of the results.

## Alcohol-Induced Flushing Among Asians

People of Asian descent consistently experience lower levels of alcoholism and higher rates of abstinence than other ethnic groups (Helzer et al. 1990; Klatsky et al. 1983). Researchers have offered two explanations for these observations (reviewed in Sue and Nakamura 1984). An “environmental” hypothesis suggests that cultural values among Asians emphasize drinking in moderation. Conversely, the “genetic” theory proposes that Asians experience a different physiological reaction to alcohol. Bridging this dichotomy, Sue and Nakamura (1984) have attributed the low rates of alcohol consumption among Asians to an interaction between cultural and physiological factors.

One physiological factor that may protect Asians from heavy drinking is the alcohol-induced flushing reaction that about one-half of Chinese, Japanese, Koreans, and other Asians experience after drinking a moderate amount of alcohol (Chan 1986). This reaction, although documented in Chinese poetry as early as the first century B.C., first was reported in the scientific literature in 1972 (Wolff 1972). The flushing reaction is characterized by a rapidly increased blood flow to the skin of the face, neck, and chest; other symptoms may include increased heart rate (i.e., tachycardia), decreased blood pressure (i.e., hypotension), headache, nausea, and vomiting (Chan 1986). The manifestations of the flushing response vary widely: Some people intensely experience the full range of symptoms, whereas others have significantly milder reactions after consuming the same alcohol dose.

## The Genetic Basis for the Flushing Reaction

**Figure 1:**
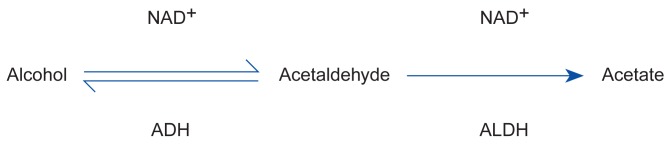
The pathway of alcohol metabolism. Once in the liver, alcohol is converted into acetaldehyde and the acetaldehyde is converted into acetate. The enzyme alcohol dehydrogenase (ADH) catalyzes the first half of alcohol metabolism, and the enzyme aldehyde dehydrogenase (ALDH) catalyzes the second half. NAD^+^ is a coenzyme that plays an accessory role in enzyme catalysis.

The alcohol-induced flushing response is associated with the process by which the body metabolizes alcohol. Two enzymes are primarily involved in alcohol metabolism—alcohol dehydrogenase (ADH), which converts alcohol to acetaldehyde, and aldehyde dehydrogenase (ALDH), which converts acetaldehyde to acetate (figure 1). Researchers hypothesize that an elevated level of acetaldehyde, a highly reactive and potentially toxic by-product, in the blood and tissue causes the flushing reaction (Harada et al. 1981). Two mechanisms could contribute to increased acetaldehyde levels: a higher-than-normal acetaldehyde production by ADH or a slower-than-normal acetaldehyde breakdown by ALDH.

### Different Forms of ADH and ALDH

Both ADH and ALDH exist in different forms (i.e., isoenzymes) in the body. Iso-enzymes are groups of enzymes that perform the same chemical reaction but have a slightly different amino acid composition and different kinetic properties.^2^ ALDH, for example, has four isoenzymes: three that primarily exist in the cell’s cytoplasm and one—called ALDH2—that is located in the cell’s energy-producing structures, the mitochondria. ALDH2 is responsible for most of the acetaldehyde breakdown in the cell. Thus, alcohol-induced flushing and other symptoms related to alcohol sensitivity have been attributed primarily to an ALDH2 deficiency (Harada et al. 1981).

**Figure 2:**
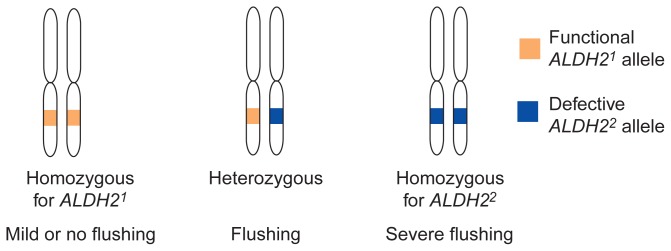
The three possible genotypes* for the *ALDH2* gene and their associated phenotypes. Each person has a pair of chromosomes carrying the *ALDH2* gene, one inherited from the mother and one from the father. The two chromosomes can carry either the same allele (homozygous) or two different alleles (heterozygous). *For a definition of this term and others used in this figure, see central glossary, pp. 182–183.

Each ALDH molecule consists of four parts, or subunits, all of which are produced (i.e., encoded) by the same gene. The gene encoding the ALDH2 isoenzyme is called *ALDH2*. This gene exists in two variants, or alleles. One allele, *ALDH2^1^*, encodes a functional enzyme subunit; the other allele, *ALDH2^2^*, contains a small change in the gene and thus encodes a defective subunit that causes ALDH deficiency. Because every person inherits two copies of each gene—one from the mother and one from the father—three different combinations of *ALDH2* alleles (i.e., three different *ALDH2* genotypes) are possible (figure 2): A person can have two *ALDH2^1^* alleles (*ALDH2^1^/2^1^* genotype), one *ALDH2^1^* allele and one *ALDH2^2^* allele (*ALDH2^1^/2^2^* genotype), or two *ALDH2^2^* alleles (*ALDH2^2^/2^2^* genotype). A person with two identical alleles (e.g., *ALDH2^1^/2^1^* genotype) is called homozygous for that allele; a person with two different alleles is called heterozygous.

The *ALDH2* genotype determines how well the ALDH2 enzyme functions: An enzyme containing any defective subunits encoded by the *ALDH2^2^* allele is less active than an enzyme containing only functional subunits encoded by the *ALDH2^1^* allele. Furthermore, an enzyme consisting only of defective subunits (i.e., in a person homozygous for *ALDH2^2^*) will be less active than an enzyme consisting of both functional and defective subunits (i.e., in a heterozygous person). As a result of the reduced enzyme activity in people with at least one *ALDH2^2^* allele, the conversion of acetaldehyde to acetate is slowed, creating excess levels of blood acetaldehyde after alcohol consumption.

### Racial Differences in the Frequency of *ALDH2* Alleles

Researchers can determine a person’s *ALDH2* genotype using DNA extracted from a small blood sample. Such molecular analyses have found that the distribution of *ALDH2* alleles varies among different ethnic groups (Goedde et al. 1992): Nearly all people of Caucasian and African-American descent are homozygous for the functional *ALDH2^1^* allele. Among Asians, however, only about 50 percent are homozygous for *ALDH2^1^*, 30 to 40 percent are heterozygous, and 5 to 10 percent are homozygous for the defective *ALDH2^2^* allele.

As described previously, these genotypes among Asians predict their response to alcohol. People who have at least one *ALDH2^2^* allele experience elevated acetaldehyde levels as well as a readily ob-servable facial flush following alcohol ingestion. Asians homozygous for *ALDH2^1^*, however, generally have no, or only a mild, flushing response.^3^

### *ALDH2* Genotype and Flushing as a Protective Factor for Alcoholism

The physiological characteristics of flushing are similar to the reactions to alcohol in people taking the medication disulfiram (i.e., Antabuse™), which is used to deter alcoholics from drinking. Molecular studies found that disulfiram mimics ALDH2 deficiency by inhibiting the enzyme and thus causing increased levels of acetaldehyde after alcohol consumption (Harada et al. 1982). The similarity between the effects of disulfiram and the flushing reaction suggests that the aversive effects of acetaldehyde may discourage drinking or excessive drinking in Asians and thus serve as a protective factor against alcoholism.

Several studies demonstrating significantly lower levels of alcohol use and alcoholism in Asians with *ALDH2^2^* alleles support this idea. For example, people homozygous for the *ALDH2^2^* allele drink very little alcohol (Takeshita et al. 1994) and are not found among alcoholics (Thomasson et al. 1991; Chao et al. 1994). Asians who are heterozygous drink significantly less and are much less likely to be alcoholic than Asians homozygous for the functional *ALDH2^1^* allele^4^ (Chao et al. 1994; Takeshita et al. 1994; Thomasson et al. 1991). The heterozygous genotype does not provide full protection from alcoholism, however, because 12 to 19 percent of Chinese and Japanese alcoholics have one *ALDH2^2^* allele (Chao et al. 1994; Thomasson et al. 1991).

Thus, a growing body of evidence suggests that possession of at least one *ALDH2^2^* allele reduces a person’s alcohol use and predisposition to alcoholism. The findings demonstrate that a genetic factor can influence, but not necessarily predetermine, the development of alcoholism. Each person’s predisposition to alcoholism probably depends on both genetic and environmental factors. Studies of alcoholics who are heterozygous for the *ALDH2* gene may allow researchers to learn more about the interactions among genes or between genes and environmental factors that affect the development of alcoholism.

### Physiological and Psychological Consequences of *ALDH* Genotype

Recent studies have investigated in more detail the physiological response to alcohol in Asians having different *ALDH2* genotypes (Wall et al. 1992, 1993, 1994; Wall and Ehlers 1995). In these studies, the subjects were male, American-born university students of Chinese, Japanese, and Korean descent whose *ALDH2* genotypes were determined from blood samples. Each subject was tested on two occasions after receiving either a placebo or 0.75 milliliters of alcohol per kilogram of body weight (mL/kg, equal to two to three alcoholic drinks). All subjects reacted comparably to the placebo beverage and had similar blood alcohol concentrations (BAC’s) after consuming the alcoholic beverage. Alcohol, however, induced very different physiological reactions in subjects, corresponding to the three different genotypes described earlier. For example, two subjects who were homozygous for the defective *ALDH2^2^* allele became ill after drink-ing the alcoholic beverage, experiencing nausea, vomiting, extreme tachycardia, and hypotension. These observations confirm the assumption that people who are homozygous for *ALDH2^2^* are most likely to experience severe physiological reactions; their intolerance of alcohol may help to explain why no one with this genotype has been found to be alcoholic.

**Figure 3:**
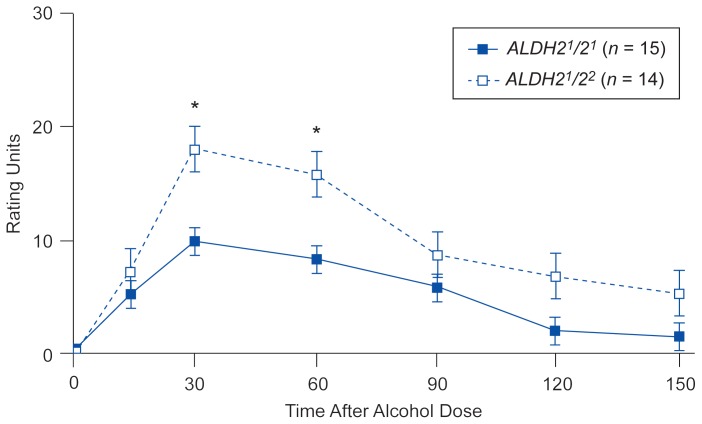
Subjective self-assessment of the “effects of alcohol” by 15 men homozygous** for *ALDH2^1^* (solid line) and 14 men with the *ALDH2^1^/ALDH2^2^* genotype (dotted line) at different times after a single dose of alcohol (0.75 ml/kg body weight). The intensity of reported alcohol effects is presented in arbitrary rating units. Error bars indicate standard error of the mean. *= significant difference between the groups (*p* < 0.05). **For further explanation, see figure 2.

The *ALDH2* genotype appears to affect not only physiological reactions but also more subjective responses to alcohol. In the experiment described above, the subjects were asked to assess the effects the alcoholic beverage had on them. The men who were heterozygous for *ALDH2* reported more intense, but not necessarily more un-pleasant, reactions to alcohol than the men with two functional *ALDH2^1^* alleles, even though they had similar BAC’s and similar recent drinking patterns. For example, heterozygous subjects reported both significantly stronger “effects of alcohol” than subjects homozygous for *ALDH2^1^* (figure 3) and more intense attributes of intoxication, such as feeling “high,” “great overall,” and “drunk.” The heterozygous subjects also tended to more strongly experience the negative effects of intoxication, such as feeling “uncomfortable,” “nauseated,” and “terrible overall,” but statistically these results were not significantly different from the responses of the subjects homozygous for *ALDH2^1^*.

These findings contradict the hypothesis that people who are heterozygous for *ALDH2* drink less because they experience only aversive alcohol effects. In fact, the results suggest that heterozygous people who regularly consume alcohol may have a more intense positive response to alcohol than those homozygous for *ALDH2^1^*. Thus, one mechanism that may contribute to the decreased rate of alcohol use and alcoholism among Asians with at least one *ALDH2^2^* allele could be heightened sensitivity to relatively low doses of alcohol (Wall et al. 1992). A person with this heightened sensitivity would need to drink less to experience the same response to alcohol as a person with lower sensitivity. Consequently, a person carrying the *ALDH2^2^* allele may avoid high intake of alcoholic beverages and thus be less likely to develop alcoholism.

If the *ALDH2* genotype affects not only people’s physiological responses but also their subjective feelings of intoxication, possession of an *ALDH2^2^* allele also may influence a person’s vulnerability to alcohol at the level of brain functioning. The following sections describe research aimed at understanding how different combinations of *ALDH2* alleles may affect the brain’s response to alcohol.

## Measuring the Brain’s Response to Alcohol

Electrophysiological techniques provide noninvasive ways to assess various elements of brain function. One of the most common methods, the electroencephalogram (EEG), is performed by attaching electrodes to a person’s scalp and recording the spontaneous electrical brain activity, known as brain waves. Brain waves can be divided into several different groups—slow alpha, fast alpha, beta, delta, and theta—according to their frequency^5^ (see table 1). Changes in brain waves (e.g., their amplitude^6^) correlate with changes in the level of consciousness or different psychological states. EEG’s allow researchers to characterize and quantify the brain’s spontaneous electrical activity and provide information about brain responses to external sensory stimuli. These responses also are called event-related potentials (ERP’s).

To measure ERP’s, the subjects are exposed to stimuli (e.g., sounds or lights) while their EEG is being recorded. When an uncommon stimulus occurs (e.g., a red light in a sequence of green and yellow lights), the brain produces a characteristic pattern of brain waves. One ERP wave that has been studied frequently in alcohol research is called P300 because it can be detected about 300 milliseconds after the uncommon stimulus. Although scientists believe that the P300 wave reflects specific neurocognitive functions (e.g., the brain’s evaluation of the stimulus and the process of selecting a response), they do not know the physiological basis for these functions.

Twin and family studies indicate that many EEG parameters are largely genetically determined (van Beijsterveldt and Boomsma 1994). A smaller number of studies also suggest moderate to high heritability for ERP’s (van Beijsterveldt and Boomsma 1994). Both EEG parameters and ERP’s are sensitive to the effects of acute and chronic alcohol use (Porjesz and Begleiter 1985).

Alcohol’s acute effects on EEG patterns have been studied since the 1930’s. In general, low doses of alcohol that result in mild intoxication and behavioral activation (less than 0.5 gram of alcohol per kilogram of body weight [g/kg], corresponding to fewer than two to three drinks) reduce the amplitudes of slow- and fast-alpha waves and increase the amplitudes of the beta waves (table 1). Moderate alcohol doses (0.5 to 1.0 g/kg) usually result in increased slow-alpha and theta waves and decreased fast-alpha waves. Larger doses of alcohol (more than 1.0 g/kg), which are associated with sedation and drowsiness, produce increases in the amplitude of the theta waves (Begleiter and Platz 1972; Ehlers et al. 1989; Kalant and Woo 1981). The magnitude of these alcohol effects, however, varies significantly among people.

The genetic vulnerability for alcoholism, as inferred from a family history of the disorder, partly determines a person’s brain response to alcohol. For example, children of alcoholics, who are at elevated risk for developing alcoholism, have a different EEG response to alcohol from control subjects without alcoholic relatives (Cohen et al. 1993; Ehlers and Schuckit 1991). Sons of alcoholic fathers in particular may have a less intense EEG response to alcohol, possibly reflecting an innate lower level of sensitivity to alcohol (Cohen et al. 1993; Ehlers and Schuckit 1991).

Not only does alcohol consumption affect the spontaneous brain activity reflected by the EEG patterns, it also alters brain responses to external stimuli (i.e., the ERP’s). Acute alcohol administration reduces the amplitude of P300 and/or increases the time between the stimulus and the appearance of P300 (i.e., the latency), suggesting reduced efficiency in brain processing (Porjesz and Begleiter 1985). The intensity of alcohol’s effect on P300 varies according to the dose ingested (Rohrbaugh et al. 1987). A person’s genetic vulnerability for alcoholism also appears to affect the P300 response. For example, alcohol-induced P300 changes were smaller in men at high risk for developing alcoholism (i.e., men having an alcoholic father) than in men at lower risk (Elmasian et al. 1982; Schuckit et al. 1988).

### ALDH2 Genotype and the Electrophysiological Response to Alcohol

Because electrophysiological responses appear to be correlated with a person’s genetic predisposition to alcoholism, researchers have used EEG patterns and ERP’s to evaluate alcohol’s effects on brain functioning in college students of Asian origin with different *ALDH2* genotypes (Wall et al. 1993; Wall and Ehlers 1995). The subjects received either a placebo or a moderate dose of 0.75 mL/kg alcohol before their brain waves were recorded. Subjects homozygous for the functional *ALDH2^1^* allele showed a typical EEG response, including increased theta and slow-alpha and decreased fast-alpha activity.

In the heterozygous subjects with one *ALDH2^2^* allele, however, the slow-alpha activity decreased significantly compared with that of the subjects homozygous for *ALDH2^1^*. This pattern of alcohol-related EEG changes in the heterozygous subjects is consistent with the increased activation that may be caused by elevated acetaldehyde levels (Wall et al. 1993) and differs from the pattern seen in Caucasian sons of alcoholics (Ehlers and Schuckit 1991). These findings suggest that EEG responses may help to measure the effects of acute intoxication on brain functioning and genetically influenced reactions to alcohol.

**Figure 4:**
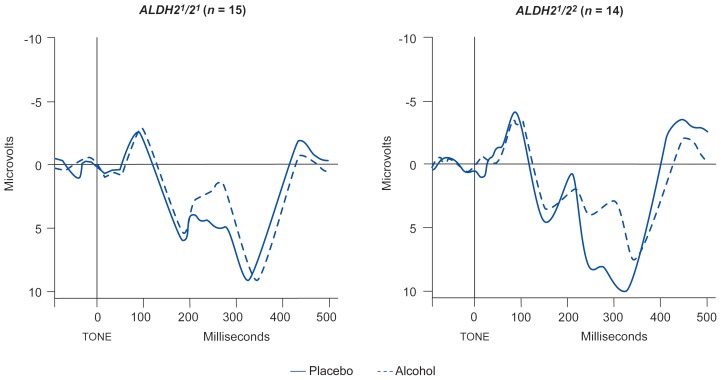
Mean event-related potentials in 15 subjects homozygous* for *ALDH2^1^* and 14 heterozygous* subjects after drinking a placebo beverage (solid line) or a beverage containing 0.75 ml/kg alcohol (dotted line). The subjects had to press a button when they heard a rarely presented tone. In both groups, alcohol delayed the appearance of the P300 wave (the segment occurring between 300 and 450 msec) and decreased P300 amplitude, but the effects were stronger in the heterozygous subjects than in the homozygous subjects. *For further explanation, see figure 2. SOURCE: Adapted from Wall and Ehlers 1995.

The response to alcohol in the same subjects also was assessed using ERP’s (Wall and Ehlers 1995). The study found that compared with a placebo, alcohol consumption in all subjects significantly decreased the amplitude and increased the latency of the P300 wave. In the heterozygous subjects, however, alcohol’s effects on P300 were significantly greater than in the subjects homozygous for *ALDH2^1^*, although all subjects had equivalent BAC’s (figure 4). These data indicate that people heterozygous for *ALDH2* experience a more intense response to alcohol and that their brain functioning may be more affected by alcohol than that of people homozygous for the *ALDH2^1^* allele.

## Theoretical and Research Implications

One model of genetic influences in alcoholism suggests that a person’s genetically determined reaction to alcohol may affect the likelihood of both drinking alcohol and developing alcoholism (Schuckit 1994). This theory predicts that compared with the general population, people at higher risk for developing alcoholism (e.g., children of alcoholic fathers) may respond less intensely to alcohol, as assessed by measures such as subjective feelings of intoxication and electrophysiological parameters. Conversely, people at lower risk for alcoholism (e.g., members of ethnic groups with a low prevalence of the disorder) would experience a heightened response to alcohol.

Studies reviewed in this article that examined the acute responses to alcohol in Asian men support this model. The men fell into three groups with respect to their genetic risk for developing alcoholism: extremely low risk (homozygous for the defective *ALDH2^2^* allele), moderately low risk (heterozygous for *ALDH2*), and relatively high risk (homozygous for the functional *ALDH2^1^* allele). The same groups were found to be highly sensitive, intermediately sensitive, and minimally sensitive to the effects of alcohol.

Although genetic factors may contribute to a person’s risk for or protection from alcoholism, their interactions with environmental factors determine their overall vulnerability. For example, as described here, the *ALDH2* genotype helps determine the level of alcohol consumption among Asians. The proposed mechanism, however, by which this gene influences the development of alcoholism (i.e., by evoking a more intense response to alcohol in brain functioning) can only manifest itself after alcohol use and may even require a certain level of alcohol consumption. In contrast, environmental factors, such as cultural attitudes that emphasize abstinence or moderate alcohol consumption, can protect against the development of alcoholism in the absence of alcohol use, when genetic factors, such as the *ALDH2* gene, likely have no influence.

No other gene has been identified that influences the level of alcohol consumption and risk for alcoholism to the same extent as *ALDH2*. Therefore, further studies of Asians with known *ALDH2* genotypes would provide a unique opportunity to improve our understanding of how the interactions between genetic and environmental factors determine a person’s risk for alcoholism. Researchers have demonstrated, for example, that alcohol use among Asian-Americans varies with the number of generations their families have lived in the United States and their level of acculturation^7^ (Chu et al. unpublished master’s thesis 1978; Sue et al. 1979). Also, substantial differences in alcohol consumption patterns and the prevalence of alcoholism exist among different ethnic groups (e.g., Chinese, Japanese, and Koreans) (Helzer et al. 1990; Kitano and Chi 1989). These observations and their relationship to differences in the genotypic distribution of *ALDH2* alleles among Asian groups warrant further investigation.

Future studies also should assess the interactions between different genes (e.g., between *ALDH2* and *ADH* genotypes) in shaping a person’s drinking behavior. Finally, genetic factors, such as *ALDH2* and their functions, have been largely unstudied in Asian women, and it is thus unclear to what extent biological or cultural differences explain gender differences in alcohol use and alcoholism prevalence among Asians. Consequently, more studies that include Asian women are needed.

Analyses of the relationships between alcohol-related genes, such as *ALDH2*, and behavior can provide insight into the determinants of alcohol-use patterns of people at various levels of genetic risk for alcoholism. These studies will contribute substantially to our knowledge of the causes of alcoholism and may help to improve alcoholism prevention and treatment. A better understanding of how the interactions of other biological and cultural factors with the *ALDH2* genotype help determine the risk of Asians for alcoholism also may have important implications for evaluating the predisposition to alcoholism of other, non-Asian populations.

## Figures and Tables

**Table 1:** Typical Effects of Different Alcohol Doses on the Activities of Different EEG Frequency Bands^1^

Frequency Band	Alcohol Dose		
	
	Low (< 0.5 g/kg) (less than 2–3 drinks)	Moderate (0.5–1.0 g/kg) (3–6 drinks)	High (> 1.0 g/kg) (more than 3–6 drinks)
			
delta (< 4 Hz)			
theta (4.0–7.5 Hz)		increased	increased
slow alpha (7.5–9.0 Hz)	decreased	increased	
fast alpha (9.0–12.0 Hz)	decreased	decreased	
beta (> 12.0 Hz)	increased		

1 The brain waves measured with an electroencephalogram (EEG) are grouped into several “bands,” each containing waves with a certain frequency range. The frequency indicates how often a wave recurs in a certain time and is measured in hertz (Hz). 1 Hz = 1 cycle per second.

SOURCES: Summarized from Begleiter and Platz 1972; Ehlers et al. 1989; and Kalant and Woo 1981.

## Footnotes

1 The terms “alcoholism” and “alcoholic” as used in this article are summary terms for the diagnoses of alcohol abuse and alcohol dependence.

2 For a definition of this and other technical terms used in this article, see central glossary, pp. 182–183.

3 The basis for this mild flushing response, which also has been reported in some other ethnic groups (e.g., Caucasians and Native Americans), is not well understood but may relate to other environmental or genetic factors, such as mutations in ADH genes.

4 Some researchers have proposed that genetic variations in alleles of ADH also could increase the rate of acetaldehyde formation, deterring heavy drinking (Takeshita et al. 1994) and contributing to decreased risk in Asians for developing alcoholism (Thomasson et al. 1991; Chao et al. 1994).

5 The frequency describes how often the wave goes through a complete cycle during a given time period. The frequency usually is measured in hertz (Hz); 1 Hz = 1 cycle per second.

6 The amplitude describes the height of a wave, from its lowest to its highest point. For electrical waves, the amplitude usually is measured in millivolts.

7 Acculturation refers to the degree to which immigrants adopt the values, attitudes, and behavior of the culture they enter.
